# Sequential azacitidine and carboplatin induces immune activation in platinum-resistant high-grade serous ovarian cancer cell lines and primes for checkpoint inhibitor immunotherapy

**DOI:** 10.1186/s12885-022-09197-w

**Published:** 2022-01-24

**Authors:** Michelle W. Wong-Brown, Andre van der Westhuizen, Nikola A. Bowden

**Affiliations:** 1 School of Biomedical Sciences and Pharmacy, University of Newcastle, Callaghan, NSW Australia; 2 Centre for Drug Repurposing and Medicines Research, University of Newcastle, Callaghan, NSW Australia; 3 Hunter Medical Research Institute (HMRI), Lot 1 Kookaburra Circuit, New Lambton Heights, Newcastle, NSW 2305 Australia; 4 Calvary Mater Newcastle, Waratah, NSW Australia; 5 School of Medicine and Public Health, University of Newcastle, Callaghan, NSW Australia

**Keywords:** Ovarian cancer, Platinum resistance, Methylation, Azacitidine, Carboplatin, Immune checkpoint inhibition

## Abstract

**Background:**

Platinum chemoresistance results in high-grade serous ovarian cancer (HGSOC) disease recurrence. Recent treatment advances using checkpoint inhibitor immunotherapy has not benefited platinum-resistant HGSOC. In ovarian cancer, DNA methyltransferase inhibitors (DNMTi) block methylation and allow expression of silenced genes, primarily affecting immune reactivation pathways. We aimed to determine the epigenome and transcriptome response to sequential treatment with DNMTi and carboplatin in HGSOC.

**Methods:**

In vitro studies with azacitidine or carboplatin alone and in sequential combination. Response was determined by cell growth, death and apoptosis. Genome-wide DNA methylation levels and transcript expression were compared between untreated and azacitidine and carboplatin sequential treatment.

**Results:**

Sequential azacitidine and carboplatin significantly slowed cell growth in 50% of cell lines compared to carboplatin alone. The combination resulted in significantly higher cell death in 25% of cell lines, and significantly higher cell apoptosis in 37.5% of cell lines, than carboplatin alone. Pathway analysis of upregulated transcripts showed that the majority of changes were in immune-related pathways, including those regulating response to checkpoint inhibitors.

**Conclusions:**

Sequential azacitidine and carboplatin treatment slows cell growth, and demethylate and upregulate pathways involved in immune response, suggesting that this combination may be used to increase HGSOC response to immune checkpoint inhibitors in platinum-resistant patients who have exhausted all currently-approved avenues of treatment.

**Supplementary Information:**

The online version contains supplementary material available at 10.1186/s12885-022-09197-w.

## Background

Standard treatment for high-grade serous ovarian cancer (HGSOC) is cytoreductive surgery followed by a combination of platinum (carboplatin or cisplatin) and taxane (paclitaxel) chemotherapies. Although initial recurrences are often platinum-sensitive, development of resistance to platinum-based chemotherapy usually occurs with subsequent recurrences [1].

When chemoresistance occurs, the disease-free period between recurrences progressively gets shorter. The most recent definition of platinum-resistant disease is disease recurrence within 6 months of completion of first-line platinum-based chemotherapy [2]. The average survival time for platinum-resistant HGSOC is approximately 9–12 months [3]. In the last decade, poly(ADP-ribose) polymerase (PARP) inhibitors (PARPi) have shown efficacy as maintenance therapy, especially in homologous recombination (HR)-deficient platinum-sensitive HGSOC [4, 5], However, two studies have shown that treatment with single agent PARPi in HR-proficient platinum-resistant HGSOC has not been very successful, with only 4–13% overall response rate (ORR) [6, 7]. It is increasingly being recognised that progression to platinum chemoresistance in ovarian cancer is associated with epigenetic changes, including increased DNA methylation and subsequent transcriptional silencing of genes [8]. These alterations affect genes implicated in treatment resistance, such as the DNA repair pathways [9], regulation of cell cycle, and of other genes associated with recognition of chemotherapy damage and apoptotic responses [10, 11]. The DNA methyltransferase (DNMT) inhibitors (DNMTi), azacitidine and decitabine passively demethylate by blocking new methylation from occurring during cell division, allowing previously silent genes and retroviral elements to be expressed [12]. Early Phase 1 and 2 clinical trials have tested DNMTi in combination with platinum chemotherapy in HGSOC. The results provided evidence that the combination can produce small incremental gains in overall survival, but not enough to be adopted into routine clinical practice [13].

Recent advances in the treatment of many solid tumours using checkpoint inhibitor (CPI) immunotherapy targeting the CTLA4 and PD1–1/PDL-1 axes held great promise for HGSOC, which is often characterised by increased tumour-infiltrating lymphocytes (TILs), a biomarker of response to CPIs. Unfortunately, the results of both monotherapy and combination CPI immunotherapy has not significantly increased progression-free or overall survival for HGSOC [14, 15]. Therefore, the major problem that remains for HGSOC treatment is the development of chemotherapy resistance [16] combined with the absence of effective further lines of treatment.

Current evidence indicates that when low-doses of DNMTi are given, methylation is blocked but the dose is not cytotoxic [17–19]. In ovarian cancer, it was shown that low-doses of DNMTi alter the epigenome and transcriptome of the tumours, which primarily affected expression of immune reactivation pathways [17]. It is currently unknown if DNMTi and carboplatin can ‘prime’ HGSOC to be more responsive to CPI immunotherapy.

Therefore, the aim of the study was to investigate if sequential DNMTi and platinum-based chemotherapy on HGSOC can increase expression of transcripts associated with immune responses, whilst occurring in parallel to decreased proliferation and/or increased cell death, therefore priming platinum-resistant HGSOC for increased response to CPI immunotherapy.

## Methods

### Cell culture and treatment

Eight ovarian cancer cell lines were used in this study; KURAMOCHI, OVSAHO, COV362, OVCAR4, COV318, TYKNU, OVKATE, and OAW28. These cell lines were confirmed by genomic and proteomic profiling to be representative of HGSOC [20]. The *BRCA* status of each cell line have been described (KURAMOCHI, OVSAHO, COV362 are *BRCA*-mutants, the others *BRCA*-wildtype) [20, 21].

The cell lines were cultured in high glucose DMEM (10% FBS) (Gibco). All cells were incubated at 37 °C with 5% CO_2_. Azacitidine (Sigma-Aldrich) and carboplatin (Sigma-Aldrich) were resuspended at 1 mM and 27 mM (10 mg/mL) respectively. All cell lines were treated with 0.5 μM azacytidine alone, 20 μM carboplatin alone and sequential combination of 0.5 μM azacitidine and 20 μM carboplatin. For azacitidine treatment, cells were treated for 72 h with 0.5 μM azacitidine, with media and azacitidine replaced every 24 h. For carboplatin treatment, cells were treated with 20 μM carboplatin for 48 h. For sequential combination azacitidine and carboplatin treatment, cell lines were first treated with 0.5 μM azacitidine for 72 h followed by 20 μM carboplatin for 48 h.

These doses of carboplatin (20 μM) and azacitidine (0.5 μM) used were lower than maximum plasma concentrations of each drug when used as chemotherapy agents [22, 23]. A maximum plasma concentration of carboplatin of 30 μM was also used as a control to treat the cells. The dose of azacitidine used in this study was also chosen as it was shown previously that lower, pharmacologically-relevant dose of DNMTi cause global demethylation [24].

### Live-cell analysis

IncuCyte® Cytotox Green was used to stain dead cells after treatment and apoptotic cells were stained with IncuCyte® Annexin Red reagents. The quantification of each population of cells was performed using IncuCyte® ZOOM (Essen BioScience). The eight cells lines were seeded in 96-well plates in triplicate wells at 20% confluency; 8000 cells/well for each cell line, 10,000 cells/well for TYKNU; and the doubling time for the cell lines ranged from 40 to 96 h. They were incubated for 24 h before treatment was added (dosage and timing described above) with Cytotox Green (final concentration of 2.5 nM) and Annexin Red (final concentration of 1:400 of the reagent stock concentration) per manufacturer’s protocol.

The wells were imaged using IncuCyte® Zoom live imaging system at 10x objective using the green and red channels (triplicate wells per treatment condition, four images per well). Phase contrast and dual-colour fluorescence images (excitation/emission settings; green 440–480/504–544 nm, red 565–605/625–705 nm) were collected for all experiments with spectral unmixing set as 8% of red removed from green) every 6 h for 7 days. The average live (cell confluence), dead (green fluorescence) and apoptotic (red fluorescence) cell count was determined from the replicated wells and data was expressed mean (green or red) confluence per mm^2^ ± SEM. Cell confluence was used as a surrogate marker of cell growth in the live-cell assay.

The normalised cell death and apoptosis was obtained by dividing the green or red fluorescence area over the phase confluence area (normalised cell death = confluence of dead (green) cells / confluence of total cells; normalised cell apoptosis = confluence of apoptotic (red) cells / confluence of total cells).

The processing definitions for analysing the confluences of phase and green and red fluorescence are described in Additional File 1.

### DNA and RNA extraction

Cell pellets were collected after 5 days of no treatment (untreated) or 5 days of sequential combination treatment (72 h with azacytidine and 48 h with carboplatin) from one independent experiment.

Cell pellets for DNA extraction were resuspended in 200 μL 1x phosphate buffered saline (Invitrogen). DNA was then extracted using the Zymo Quick-DNA Miniprep Kit (Zymo Research) per manufacturer’s protocol. DNA was quantified using the Qubit™ DNA Broad Range Assay Kit (Invitrogen) and purity assessed by 260/280 ratios (> 1.8) using the Epoch Microplate Spectrophotometer (BioTek). DNA integrity was analysed using the TapeStation and D1000 ScreenTape Assay. Cell pellets with a minimum of 250 ng genomic DNA were used for methylation array.

Total RNA was extracted by TRIzol (Invitrogen) extraction method according to standard laboratory protocol. RNA was quantified using the Qubit™ RNA High Sensitivity Assay Kit (Invitrogen) and purity assessed by 260/280 ratios (> 1.8) as above. RNA integrity was analysed using the TapeStation and RNA ScreenTape Assay (Agilent Technologies). Cell pellets with a minimum of 1 μg total RNA and RNA Integrity Number (RIN) ≥8 were used for RNA sequencing.

In total, four HGSOC cell lines, COV362, OVCAR4, TYKNU, and OAW28 fulfilled the minimum requirements for methylation array and RNA sequencing to be performed. Additionally, these 4 cell lines show the most significant changes in cell confluence and relative cell death and apoptosis compared to standard-of-care platinum treatment (Additional Files 2, 3, and 4).

### Methylation array

Microarray hybridisation was performed by the Australian Genome Research Facility (AGRF). Genomic DNA extracted from cell lines as above were bisulfite-treated using the EZ DNA Methylation Kit (Zymo Research). Genome-wide DNA methylation profiles were obtained using the Infinium MethylationEPIC Beadchip (Illumina), which interrogates over 850,000 methylation sites across the genome.

Data preprocessing and analyses were performed in the statistical programming environment R v3.6.2 with the package Chip Analysis Methylation Pipeline for Illumina HumanMethylation450 and EPIC (ChAMP) v3.11 [25]. Probes that had a detection *P*-value> 0.01, non-CpG probes, and SNP-related probes were removed from the analysis. Probes on the X and Y chromosomes were excluded from the analysis. Normalisation and background correction were applied to the methylation data using PBC within the ChAMP package.

Differentially methylated probes (DMPs) were identified using the package within ChAMP, which calculated the difference in β-value between the untreated and azacitidine and carboplatin-treated groups. The β-value is the ratio of intensities between methylated and unmethylated alleles, with β being between 0 (unmethylated) and 1 (fully methylated) [26]. The DMPs were annotated according to its position relative to nearby CpG island and transcription start site (TSS) based on the Infinium MethylationEPIC manifest. CpG islands were defined as DNA regions longer than 500 bases containing more than 55% GC content and observed-to-expected CpG ratio greater than 40%. CpG shores were defined as 2 kb regions immediately upstream and downstream of CpG islands. CpGs were considered to be associated with transcripts if they mapped to the region of 200–1500 bases upstream of TSS (TSS1500), within 200 bases upstream of TSS (TSS200), 5’UTR, 1st exon, gene body, or 3’UTR. The Infinium MethylationEPIC Beadchip data are available for download at the Gene Expression Omnibus (GEO) data repository (GSE168225). To correct *P* values for multiple hypothesis testing, false discovery rate (FDR) was calculated using Benjamini-Hochberg correction, and methylation sites in CpG sites/regions with FDR-adjusted *P*-value< 0.275 were considered statistically significant.

The Genomic Regions Enrichment of Annotations Tool (GREAT) (http://great.stanford.edu/) was used to analyse the functional significance of differentially methylated regulatory regions [27].

### RNA-Seq

Whole transcriptome sequencing (RNA-Seq) was performed by AGRF. Total RNA was extracted from cell lines and quality of the total RNA evaluated as above. Briefly, the ribosomal RNA (rRNA) was removed from the extracted total RNA by Illumina Ribo-Zero Plus rRNA Depletion Kit (Illumina), and strand-specific RNA-seq libraries were built by TruSeq Stranded Total RNA (Illumina) according to the manufacturer’s protocol. Quality control of the libraries was performed by Agilent 2100 Bioanalyzer (Agilent Technologies). RNA sequencing was performed on the Illumina NovaSeq 6000 platform (Illumina), and 100 bp paired-end reads were generated, with a sequencing depth of 30-60 M reads. This is sufficient for characterisation of gene expression levels.

The RNA-Seq reads were first trimmed with Cutadapt to remove adapter sequences, primers, poly-A tails and low quality reads. The reads were aligned to a reference genome GRCh37/hg19 using the sequence alignment tool HISAT2. Counts for mapped reads for genomic features such as genes, exons, promoters, gene bodies, genomic bins, and chromosomal locations were created using featureCounts.

Differential expression analysis between the untreated and treatment groups was performed using the DESeq2 package v3.12. The estimation of transcript abundances is based on normalised base counts from all samples, dividing by size factors. The biological processes altered by gene expression changes between the groups were assessed using Ingenuity Pathway Analysis (IPA). The RNA sequencing data is available for download at the GEO repository (GSE169617).

## Results

### Combination of azacitidine and carboplatin decreases ovarian cancer cell growth

**Fig. 1:**
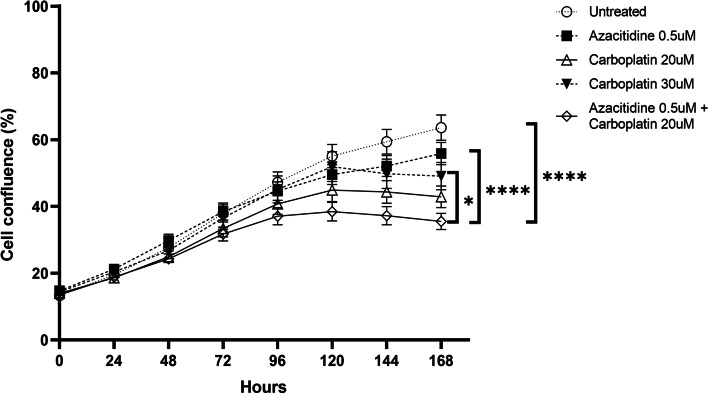
Average proliferation over time for ovarian cancer cells treated with azacitidine, carboplatin and combination azacitidine and carboplatin. Data displayed is mean ± SEM of triplicates of three independent experiments for 8 cell lines

Cellular confluence was measured to determine if ovarian cancer cell growth is affected by combined azacitdine and carboplatin. The sequential combination of azacitidine and carboplatin significantly slowed the growth of all cell lines (Fig. 1). Cells treated with azacitidine or carboplatin only grew at a non-significant slower rate compared to untreated control. This suggests that sequential azacitidine and carboplatin can decrease the rate of cell growth, even if they do not significantly result in a large increase in apoptosis and cell death.

**Fig. 2:**
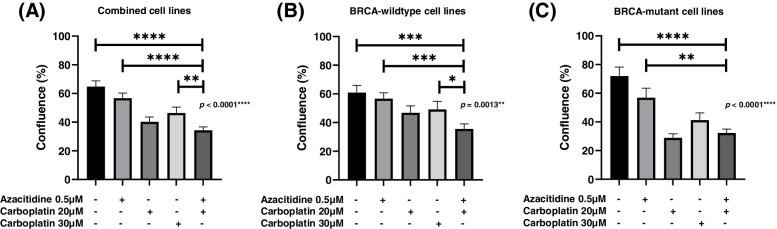
Percentage confluence 7-days after treatment with azacitidine, carboplatin or combination azacitidine and carboplatin in (**A**) all cell lines, (**B**) *BRCA*-wildtype cell lines, and (**C**) *BRCA-*mutant cell lines. Data displayed is mean ± SEM of triplicates of three independent experiments

Combined analysis of all cell lines showed that cell confluence was lower compared to single treatment of azacitidine or carboplatin only, and significantly lower than single treatment of higher dose carboplatin (30 μM) (Fig. 2A). This was also observed in cell lines that did not have *BRCA* mutations (Fig. 2B). In *BRCA*-mutant cell lines, cell confluence was lower compared to single treatment of azacitidine or higher-dose carboplatin only (Fig. 2C).

The most significant differences in cell confluence between treatment groups occurred 7 days after commencement of treatment (ANOVA Multiple Corrections Test *p* < 0.05). For KURAMOCHI, COV362, OVCAR4, and TYKNU, confluence was significantly lower when treated with combined azacitidine and low-dose carboplatin compared to a single treatment of higher dose carboplatin (Additional File 2).

### Sequential azacitidine and carboplatin increases cell death

**Fig. 3:**
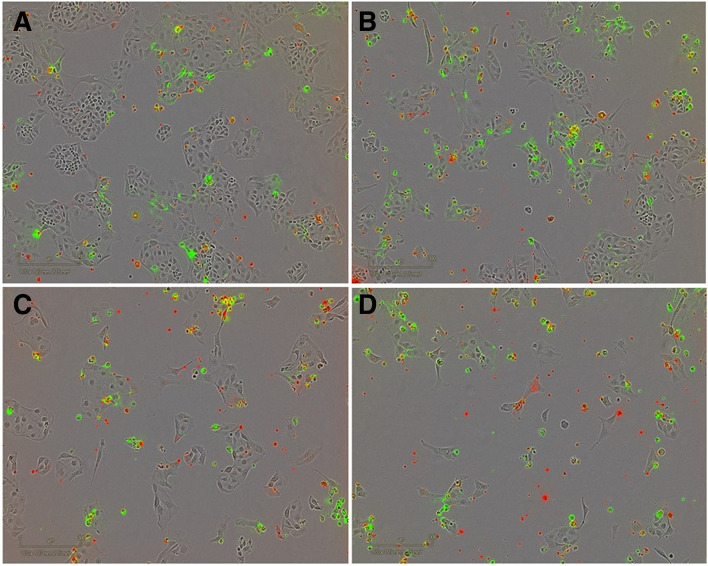
Cell death and apoptosis in ovarian cancer cell lines 7-days after treatment with azacitidine, carboplatin, or combined treatment. Cytotox (cell death; green), Annexin V (apoptosis; red), overlap between Cytotox and Annexin V (yellow) (**A**) No treatment; (**B**) 0.5 μM azacitidine (**C**) 20 μM Carboplatin; (D) 0.5 μM azacitidine and 20 μM carboplatin

To investigate if sequential azacitidine and carboplatin results in increased cell death, apoptosis and cell death were quantified in real-time following single agent azacitidine or carboplatin, and sequential combination treatment (Fig. 3).

**Fig. 4:**
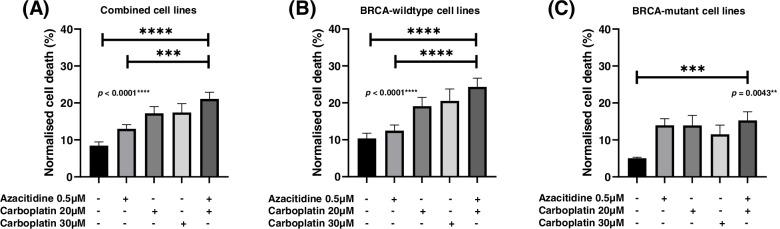
Percentage cell death in ovarian cancer cells 7-days after azacitidine, carboplatin or combination azacitidine and carboplatin treatment in (**A**) all cell lines, (**B**) *BRCA*-wildtype cell lines, and (**C**) *BRCA-*mutant cell lines. Percentage cell death is represented as the mean ± SEM of triplicates of three independent experiments

**Fig. 5:**
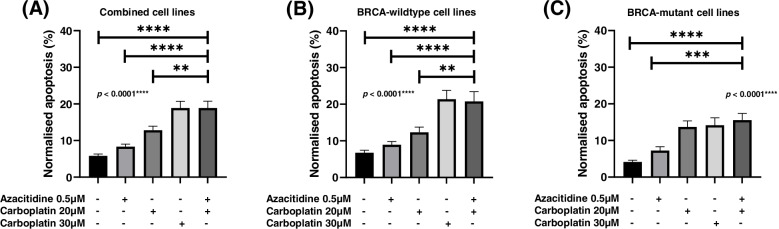
Percentage apoptosis in ovarian cancer cells 7-days after azacitidine, carboplatin or combination azacitidine and carboplatin treatment in (**A**) all cell lines, (**B**) *BRCA*-wildtype cell lines, and (**C**) *BRCA-*mutant cell lines. Percentage apoptosis is represented as the mean ± SEM of triplicates of three independent experiments

Azacitidine or carboplatin treatment slightly increased cell death and apoptosis compared to untreated cell lines. However, the sequential azacitidine and carboplatin treatment induced greater cell death and apoptosis compared to either single treatment alone (Figs. 4 and 5).

Combined analysis of all cell lines showed a trend towards higher cell death that did not reach significance compared to single treatment of low-dose (20 μM) or higher-dose (30 μM) carboplatin only (Fig. 4A). This was also observed in cell lines with and without *BRCA* mutations (Fig. 4B and C). All cell lines except OVCAR4 showed significant differences in cell death between treatment groups (ANOVA Multiple Corrections Test *p* < 0.05). For TYKNU, combination azacitidine and carboplatin showed significantly higher cell death compared to the higher dose of carboplatin (30 μM) (Additional File 3). Importantly, this suggests that it may be possible to utilize a lower dose of 20 μM carboplatin in combination with azacitidine to increase ovarian cancer cell death.

Combined analysis of all cell lines showed that cell apoptosis was significantly higher after sequential azacitidine and carboplatin compared to single treatment of azacitidine or low-dose carboplatin (20 μM) (Fig. 5A). This was also observed in *BRCA*-wildtype cell lines (Fig. 5B). In *BRCA-*mutant cell lines, cell apoptosis showed a trend towards being higher than single treatment of carboplatin (Fig. 5C). All cell lines except TYKNU showed significant differences in normalised cell apoptosis between treatment groups (ANOVA Multiple Corrections Test *p* < 0.05). One cell line, OVCAR4 had significantly higher cell apoptosis after combined azacitidine and carboplatin compared to higher-dose of 30 μM carboplatin. Two cell lines, COV318 and OAW28 had significantly higher cell apoptosis when treated with combined azacitidine and carboplatin compared to treatment with low-dose carboplatin (20 μM) (Additional File 4). Taken together, this data indicates sequential azacitidine and carboplatin induces at least an equivalent or greater amount of apoptosis and cell death as either treatment alone.

### Differential methylation in response to combination of azacitidine and carboplatin

To investigate the potential for sequential azacitidine and carboplatin to induce biological pathways related to CPI sensitivity, global demethylation was assessed in untreated cells compared to cells treated with sequential azacitidine and carboplatin. The reference genome used in all analyses was GRCh37/hg19. Probe filtering resulted in 711,405 probes after removing those that failed with a detection *P* > 0.01 in more than one sample and probes that are not represented by a minimum of three beads on the array.

Differential methylation was assessed in CpG sites (DMPs); *P* < 0.05 after multiple comparison correction FDR = 0.275 was considered significant. In total, there were 86,979 DMPs found to be significantly different between the untreated and sequential azacitidine and carboplatin groups (*P* < 0.05, FDR-adjusted *P* < 0.275 and difference in β-value (Δβ) ≥ 0.1).

Out of the 86,979 DMPs, 86,966 were hypomethylated in the treatment group, and 36,764 DMPs were associated with a gene. Gene ontology analysis of the 36,764 DMPs was performed using the annotation tool GREAT by associating the genomic regions to a single nearby gene [27]. One of the biological processes that was significantly enriched in response to combination azacitidine and carboplatin treatment was the regulation of intrinsic apoptotic signaling pathway (*p* = 3.55e-37), the main mechanism of programmed cell death after treatment with carboplatin (Additional File 5).

### Differential expression of genes involved in response to combination of azacitidine and carboplatin

To investigate differentially expressed genes after combined azacitidine and carboplatin, RNA sequencing was performed on the four cell lines with adequate RNA yield and integrity before and after combination treatment. Out of the 24,701 genes differentially expressed between cells that were untreated and treated with combined azacitidine and carboplatin, 1383 genes showed significant difference in expression between the untreated and treatment groups (*p* < 0.5). 36 annotated genes were upregulated after combination treatment compared to the untreated group (fold change> 1.5, *p <* 0.5). Analysis by IPA was performed to investigate the pathways significantly upregulated by the combination azacitidine and carboplatin treatment. One of the most significantly upregulated pathways after combination treatment was the apoptosis signaling pathway (*p* = 1.03e-02), which is triggered to respond to carboplatin-induced DNA damage (Additional File 6). This indicates an increase in apoptotic cell death response, also shown in the gene ontology analysis of the significantly hypomethylated DMP-associated genes.

Three of the most highly enriched were pathways related to apoptotic programmed cell death (apoptotic signaling, (*p* = 1.03e-02), TNFR1 signaling, (*p* = 2.5e-03), death receptor signaling (*p* = 8.72e-03)), which are involved in response to chemotherapy. Also of note was the enrichment of the antigen presentation pathway, which is essential in presenting neoantigens or foreign peptide antigens to trigger T cell immune response [28]. Similarly, three of the most significantly upregulated pathways are related to immune and proinflammatory responses (cytotoxic T lymphocyte-mediated apoptosis of target cells, TWEAK signaling, TNFR1 signaling) (Additional File 6).

### Integrated methylome-transcriptome analysis

**Fig. 6:**
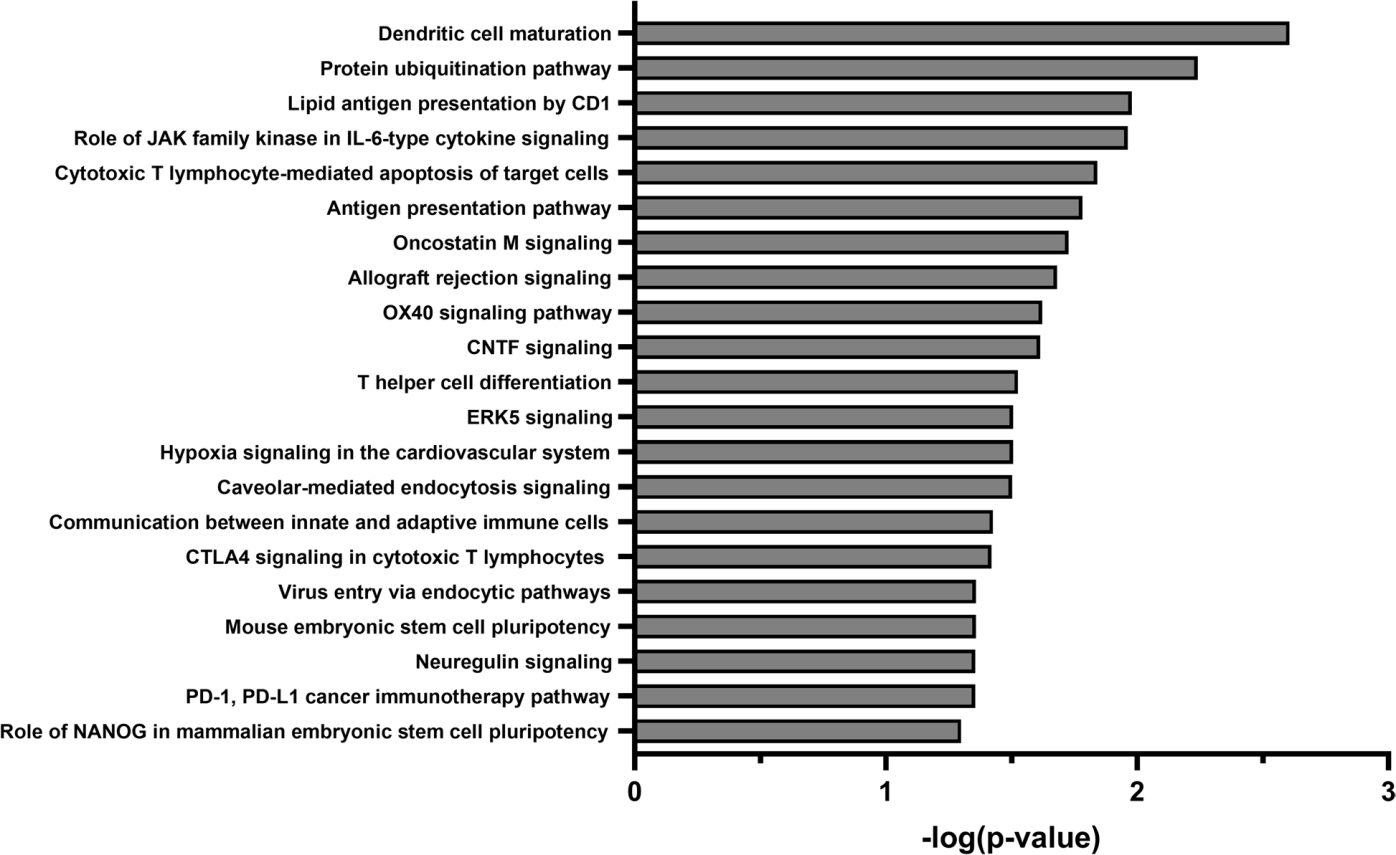
The pathways enriched by hypomethylated and upregulated genes (fold change ≥1.5, *p*-value < 0.5)

An integrated methylome-transcriptome analysis was performed to examine the association between CpG methylation and gene expression in the tumour cells after combination treatment. The DMPs obtained from the TSS1500 and TSS200 regions were selected for subsequent analysis as promoters and their surrounding regions are primarily responsible for gene expression regulation. We focused on promoter CpG island-containing genes that were simultaneously hypomethylated and upregulated after combined azacitidine and carboplatin treatment, and ten genes were identified (fold change≥1.5, *p*-value< 0.5). The network constructed from the ten upregulated genes indicated that the majority of changes were in pathways relating to immune responses, including cytotoxic T lymphocyte-mediated apoptosis (*p* = 1.44e-02), antigen presentation pathway (*p* = 1.65e-02), CTLA4 signaling in cytotoxic T lymphocytes (*p* = 3.79e-02), and PD-1 and PD-L1 cancer immunotherapy (*p* = 4.42e-02) (Fig. 6). Our integrated analysis of the methylome and transcriptome suggests that combined azacitidine and carboplatin may activate immune responses to immune checkpoint inhibitors. Addditionally, one of the genes shown to be significantly hypomethylated and upregulated after combined azacitidine and carboplatin treatment was β2 microglobulin (β2m) (fold change = 5.319, *p* = 0.203), which is involved in many of these immune-related pathways.

## Discussion

The aim of the study was to investigate the effect of sequential DNMTi and platinum-based chemotherapy on HGSOC and to determine if an increase in expression of transcripts associated with immune responses occurs in parallel to decreased proliferation and/or increased cell death, therefore priming platinum-resistant HGSOC for increased response to CPI immunotherapy.

There have been several clinical trials aiming epigenetically prime for resensitization of platinum-resistant ovarian tumours to platinum therapy, with overall response rates of 15–35% in a heavily pretreated cohort [17, 18, 29, 30]. Another Phase 2 clinical trial showed that epigenetic priming in combination with carboplatin did not significantly increase progression-free survival compared to standard non-platinum chemotherapy in platinum-resistant ovarian cancer [31]. However, analysis of the patient samples from these trials showed the upregulation of pathways involved in immune reactivation pathways [17], suggesting that this treatment combination may be able to prepare the ovarian tumours for treatment with CPI immunotherapy.

Sequential treatment of HGSOC cell lines with low-dose azacitidine and carboplatin was effective in slowing cell growth and increasing cell death and apoptosis. When compared to single treatment of azacitidine or carboplatin the sequential combination induced equivalent or higher levels of cell death and apoptosis. The effect of lower doses of DNMTi is DNA demethylation and the reexpression of silenced genes, possibly those associated with cell proliferation, DNA repair, apoptosis, and senescence. At higher doses of DNMTi, DNMT is trapped to DNA and forms covalent adducts [32, 33], triggering DNA damage response and subsequent cytotoxicity. Current evidence indicates that when low-doses of DNMTi are given, methylation is blocked but the dose is not cytotoxic [17–19]. The repetitive low-dose administration of DNMTi may result in longer exposure to the active metabolites, subsequently causing sustained demethylating effects [13]. In ovarian cancer, repetitive low doses of DNMTi was shown to alter the epigenome and transcriptome of the tumours, which primarily affected expression of immune-related and DNA repair pathways [17]. Our approach of sequential treatment with repetitive low-dose azacitidine followed by carboplatin is based on the understanding of these studies, which is to induce DNA damage by platinum chemotherapy after upregulating expression of DNA damage response transcripts by inhibiting DNA methylation. This combination may cause the cells to accumulate further mutations, become stressed and possibly induce new apoptotic pathways. The increased tumour mutation burden and altered transcriptome is predictive of a positive response to immunotherapy.

Cytotoxic agents are typically administered at lower doses in combination therapy compared to single-drug therapy to reduce toxic treatment side effects [34]. Previous studies have shown that the combination of low-dose azacitidine with dose-reduced carboplatin was well-tolerated, the most common adverse effects being fatigue and myelosuppression and 6.6% with Grade 4 neutropenia or thrombocytopenia [35]. A Phase 1 clinical trial of combined guadecitabine, a DNMTi, with carboplatin also showed that the most common adverse effects were fatigue and myelosuppression. However, in this study, 50% of patients had ≥ Grade 3 neutropenia in the group treated with DNMTi and dose-reduced carboplatin, compared to 100% of patients in the cohort treated with DNMTi and standard carboplatin dose, with an overall response rate of 15% [13]. This indicates that a lower dose of carboplatin is able to cause tumour death in combination with azacitidine, and may be used instead of the higher clinically-utilised dose of carboplatin in heavily pre-treated HGSOC.

The antigen presentation process is tightly regulated and often blocked by tumour cells via immune checkpoints such as CTLA4 and PD-1, suggesting that the upregulation of antigen presentation is indicative of response to immune checkpoint inhibitors. Our integrated methylome-transcriptome pathway analysis showed enrichment of the CTLA4 signaling and PD-1 and PD-L1 cancer immunotherapy pathways coupled with an increase in antigen presentation and major histocompatibility complex (MHC) class I after sequential azacitidine and carboplatin treatment of HGSOC cell lines.

The integrated methylome-transcriptome analysis also showed hypomethylation and upregulation of β2m after combined azacitidine and carboplatin. β2m is a component of MHC class I heterodimers responsible for antigen presentation, which shuttles peptide fragments to the cell surface for recognition by CD8+ T lymphocytes [36, 37]. In ovarian cancer, an oncolytic virus-based therapy was shown to efficiently kill the cells and induce overexpression of β2m [38]. High expression of β2m in ovarian tumours is also associated with overexpression of HLA-E, a non-classical MHC class I molecule [39]. In turn, the overexpression of HLA-E was correlated with expression of tumour-infiltrating CD8+ T lymphocytes [39]. The upregulation of β2m after combined azacitidine and carboplatin in this study may therefore indicate a trend towards sensitivity to CPI immunotherapy.

Recent advances in the treatment of solid tumours using CPI immunotherapy has only resulted in modest improvement to overall response rates in HGSOC. Two clinical trials of CPI immunotherapy in patients with platinum-resistant recurrent ovarian cancer showed objective response rate of only 9.6% (12/25) for anti-PD-L1 and 9% (11/97) for anti-PD-1 [15, 40]. Preclinical studies have shown that epigenetic therapies can enhance anti-tumour response to PD-1 or PD-L1 inhibition. Similar to our study, Yu et al. showed that treatment with low-dose decitabine resulted in the upregulation of immune-related genes, including those involved in antigen-processing and antigen-presenting, in colorectal tumour-bearing mice [41]. Tumours treated with low-dose decitabine also had greater CD4^+^ and CD8^+^ PD-1-positive tumour-infiltrating T-lymphocytes in the tumour microenvironment compared to the untreated tumours, suggesting an enhanced response to PD-1 inhibition [41]. A recent study using DNMTi and other epigenetic inhibitors showed a decrease in tumour burden and increased survival in ovarian cancer. This combination upregulated type I interferon response and MHC class I and PD-L1 on the tumour cell surface and increased PD-1 CD4^+^ and CD8^+^ T-cells [42], indicative of a possible response to immunotherapy. A recent Phase 2 clinical trial using DNMTi to prime platinum-resistant ovarian cancer to respond to CPI immunotherapy resulted in a response rate of 54.6% [43]. Our hypothesis of using the combination of epigenetic treatment with standard-of-care platinum chemotherapy to prime platinum-resistant ovarian cancer to respond to CPI immunotherapy shows similar increase in immune-related pathways.

The results from this study are consistent with our previous study in melanoma, in which the sequential combination of a DNMTi and platinum chemotherapy induced greater cell death and slowed cell proliferation [24]. Several studies have shown that while DNMTi can resensitize ovarian cancer to platinum chemotherapy, the combination can also cause the reactivation of immune pathways [17, 18]. Our findings correlate with these previous studies in our aim to show that the pathways that are demethylated and upregulated by combined azacitidine and carboplatin are related to immunomodulatory responses, and the combination may increase response to immune checkpoint inhibitor therapies. CPI monotherapy has previously been shown to result in hyperprogressive disease, which is a rapid increase in tumour growth and metastatic spread after CPI administration. This was observed in clinical trial studies in 11–29% of patients with non-small cell lung, head and neck squamous cell, and gastric cancers [44–47]. Our results indicate that in the ‘priming’ stage with sequential azacitidine and carboplatin, there is decrease in cell growth, suggesting that the ovarian tumour progression may be controlled with this combination before further treatment with CPI immunotherapy.

There are limitations to our cell line model-based study, which lacks tumour heterogeneity and microenvironment. It would be beneficial to replicate this study in an in vitro 3D cultures from primary tumours, ascites, or metastases, especially from platinum-resistant patients, to model the ovarian cancer tumour microenvironment [48]. Additionally, we will need to validate the demethylated and upregulated genes at the protein expression level by Western blotting. A clinical trial will be required to observe the potential of combined sequential azacitidine and carboplatin treatment to prime platinum-resistant ovarian cancer patients to respond to CPI immunotherapy. The combination of DNMTi and platinum therapy has is already being tested in clinical trials with platinum-resistant ovarian cancer patients [13, 29, 30]. The translation of our study into a clinical trial will be feasible due to the existing evidence for safety and pharmacokinetics of azacitidine and carboplatin as they have been approved for clinical usage and the combination can be repurposed for use in platinum-resistant ovarian cancer patients. We will be able to monitor the expression of the tumour immune markers after combined sequential azacitidine and carboplatin to sensitize to CPI immunotherapy in melanoma patients enrolled in an investigator-initiated Phase 2 clinical trial from our research group (ACTRN12618000053224).

## Conclusion

The results of this study indicate that sequential azacitidine and carboplatin can slow tumour cell growth, and sequential combined low doses of azacitidine and carboplatin can slightly increase HGSOC cell death compared to clinically-relevant dose of carboplatin, which is an important outcome especially in platinum-resistant disease. The combination of sequential azacitidine and carboplatin treatment demethylates and upregulates pathways that are involved in immune response and immune checkpoint blockade. Taken together, the results suggest that the addition of azacitidine to standard-of-care carboplatin can be used in increase HGSOC response to immune checkpoint inhibitors.

## Supplementary Information

**Additional file 1: Supplementary Table 1**: Suggested processing definitions using the Incucyte® Zoom.

**Additional file 2.**

**Additional file 3.**

**Additional file 4.**

**Additional file 5.**

**Additional file 6.**

## Data Availability

The data that support the findings of this study are openly available in Gene Expression Omnibus (GEO) at https://www.ncbi.nlm.nih.gov/geo/, reference number GSE168225 and GSE169617.

## Abbreviations

CPI: Checkpoint inhibitor
CTLA-4: Cytotoxic T-lymphocyte-associated protein 4
DMEM: Dulbecco’s Modified Eagle Medium
DMP: Differentially methylated probes
DNMTi: DNA methyltransferase (DNMT) inhibitor
FDR: False discovery rate
HGSOC: High-grade serous ovarian cancer
MHC: Major histocompatibility complex
PD-1: Programmed cell death protein 1
PD-L1: Programmed death-ligand 1
TIL: Tumour infiltrating lymphocyte
TSS: Transcription start site
