# Supplementary material for: Sequential azacitidine and carboplatin induces immune activation in platinum-resistant high-grade serous ovarian cancer cell lines and primes for checkpoint inhibitor immunotherapy
Source: BMC Cancer. 2022 Jan 24;22:100. doi: 10.1186/s12885-022-09197-w (PMC8787901; doi:10.1186/s12885-022-09197-w)
Supplement: Supplementary file 1 — Additional file 1: Supplementary Table 1: Suggested processing definitions using the Incucyte® Zoom. [file 12885_2022_9197_MOESM1_ESM.docx]

Supplementary Table 1: Suggested processing definitions using the Incucyte® Zoom.

| **Cell line** | **Parameter** | **Radius (µm)** | **Adjustment** | **Edge** | **Fill (µm)** | **Area (µm)** | **Eccentricity** | **Mean intensity** | **Integrated intensity** |
| --- | --- | --- | --- | --- | --- | --- | --- | --- | --- |
| KURAMOCHI Cytotox | Adaptive | - | 2.0 | 8 | 0 | >100, <1000 | <0.8 | 0 | 0 |
| KURAMOCHI Annexin V | Adaptive | - | 2.0 | 35 | 2000 | 0 | 0 | 0 | 0 |
| OVSAHO Cytotox | Top-Hat | 40.0 | 1.0 | 42 | 0 | >100 | 0 | >20 | 0 |
| OVSAHO Annexin V | Adaptive | - | 1.5 | 20 | 0 | 0 | 0 | 0 | 0 |
| COV362 Cytotox | Top-Hat | 40.0 | 2.0 | 30 | 0 | >100 | >50 | 0 | 0 |
| COV362 Annexin V | Adaptive | - | 1.0 | 20 | 0 | 0 | 0 | 0 | 0 |
| OVCAR4 Cytotox | Adaptive | - | 0.5 | 20 | 0 | 0 | 0 | >100 | 0 |
| OVCAR4 Annexin V | Adaptive | - | 0.2 | 32 | 400 | 0 | 0 | 0 | 0 |
| COV318 Cytotox | Adaptive | - | 2.0 | 38 | 0 | >100, <1200 | 0 | 0 | 0 |
| COV318 Annexin V | Adaptive | - | 2.0 | 30 | 0 | 0 | 0 | 0 | 0 |
| TYKNU Cytotox | Adaptive | - | 2.0 | 32 | 0 | >100, <1000 | 0 | >50 | 0 |
| TYKNU Annexin V | Adaptive | - | 0.5 | 25 | 0 | 0 | 0 | 0 | 0 |
| OVKATE Cytotox | Adaptive | - | 2.0 | 38 | 100 | >150 | 0 | 0 | 0 |
| OVKATE Annexin V | Adaptive | - | 1.0 | 20 | 0 | 0 | 0 | 0 | 0 |
| OAW28 Cytotox | Adaptive | 1.0 | - | -6 | 200 | 0 | 0 | >100 | 0 |
| OAW28 Annexin V | Adaptive | 0.5 | - | 18 | 0 | 0 | 0 | 0 | 0 |
