# Supplementary figures and images for: Sequential azacitidine and carboplatin induces immune activation in platinum-resistant high-grade serous ovarian cancer cell lines and primes for checkpoint inhibitor immunotherapy
Source: BMC Cancer. 2022 Jan 24;22:100. doi: 10.1186/s12885-022-09197-w (PMC8787901; doi:10.1186/s12885-022-09197-w)

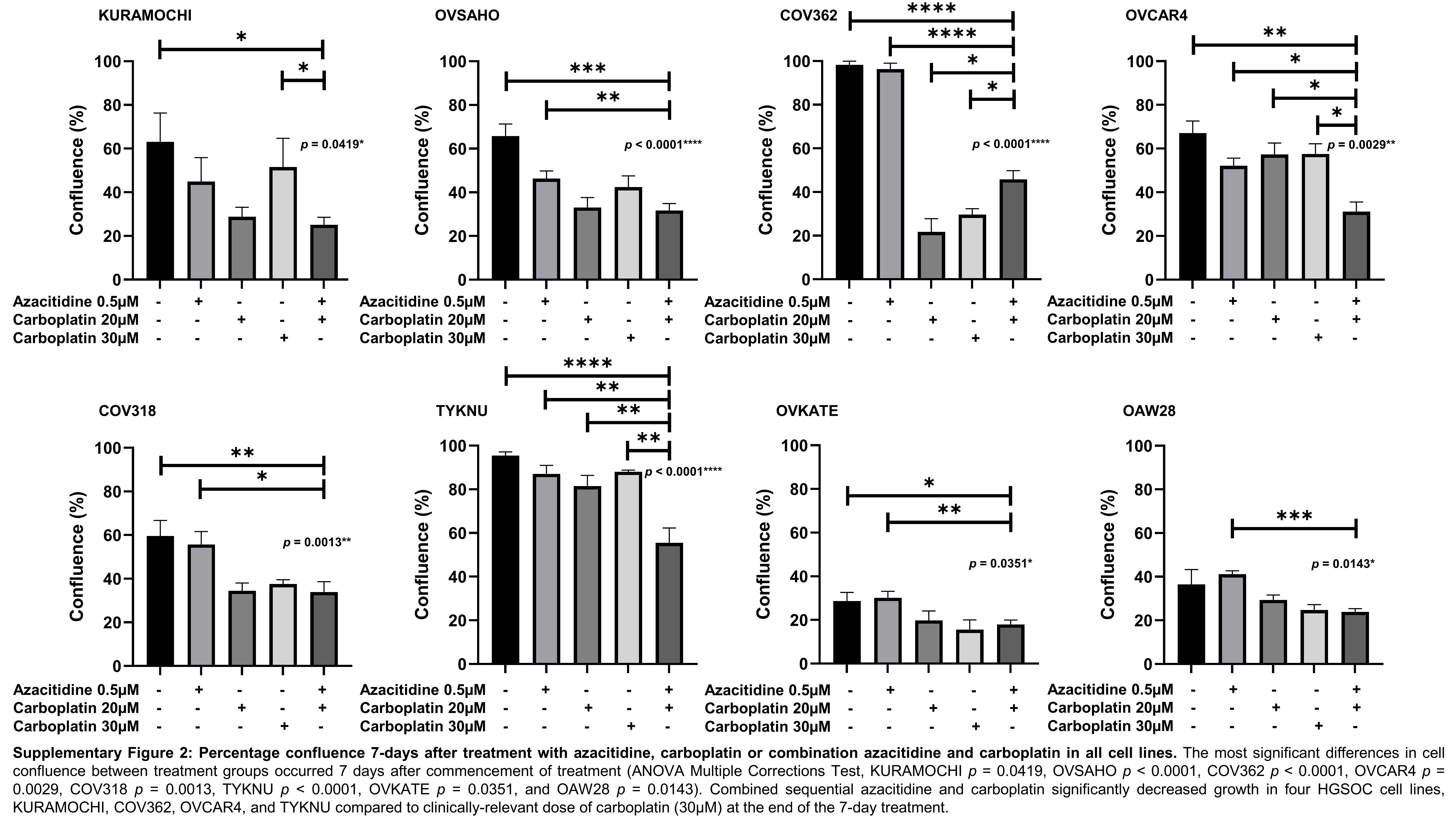

Supplement: Supplementary file 2 — Additional file 2. [file 12885_2022_9197_MOESM2_ESM.tif]

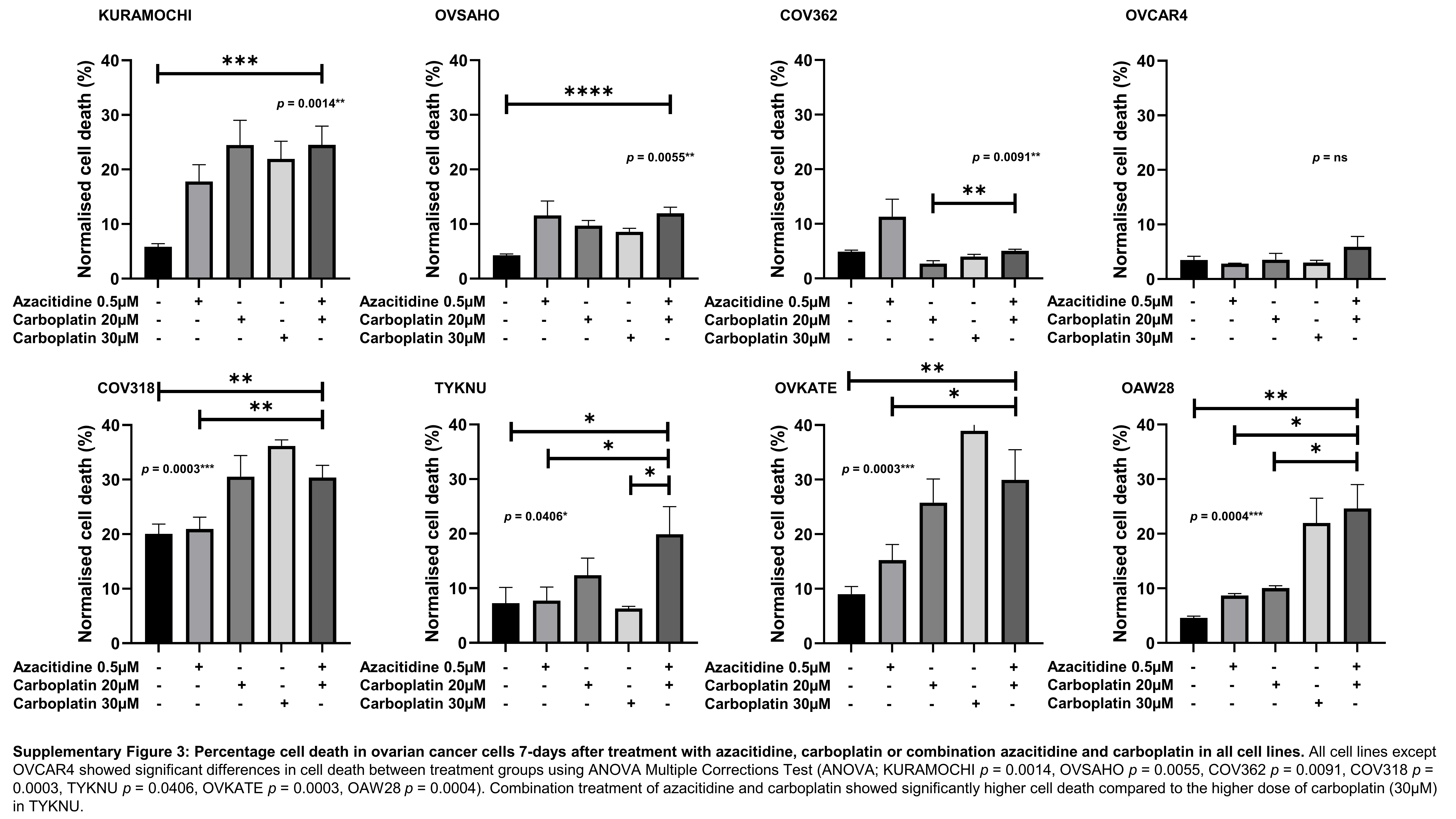

Supplement: Supplementary file 3 — Additional file 3. [file 12885_2022_9197_MOESM3_ESM.tif]

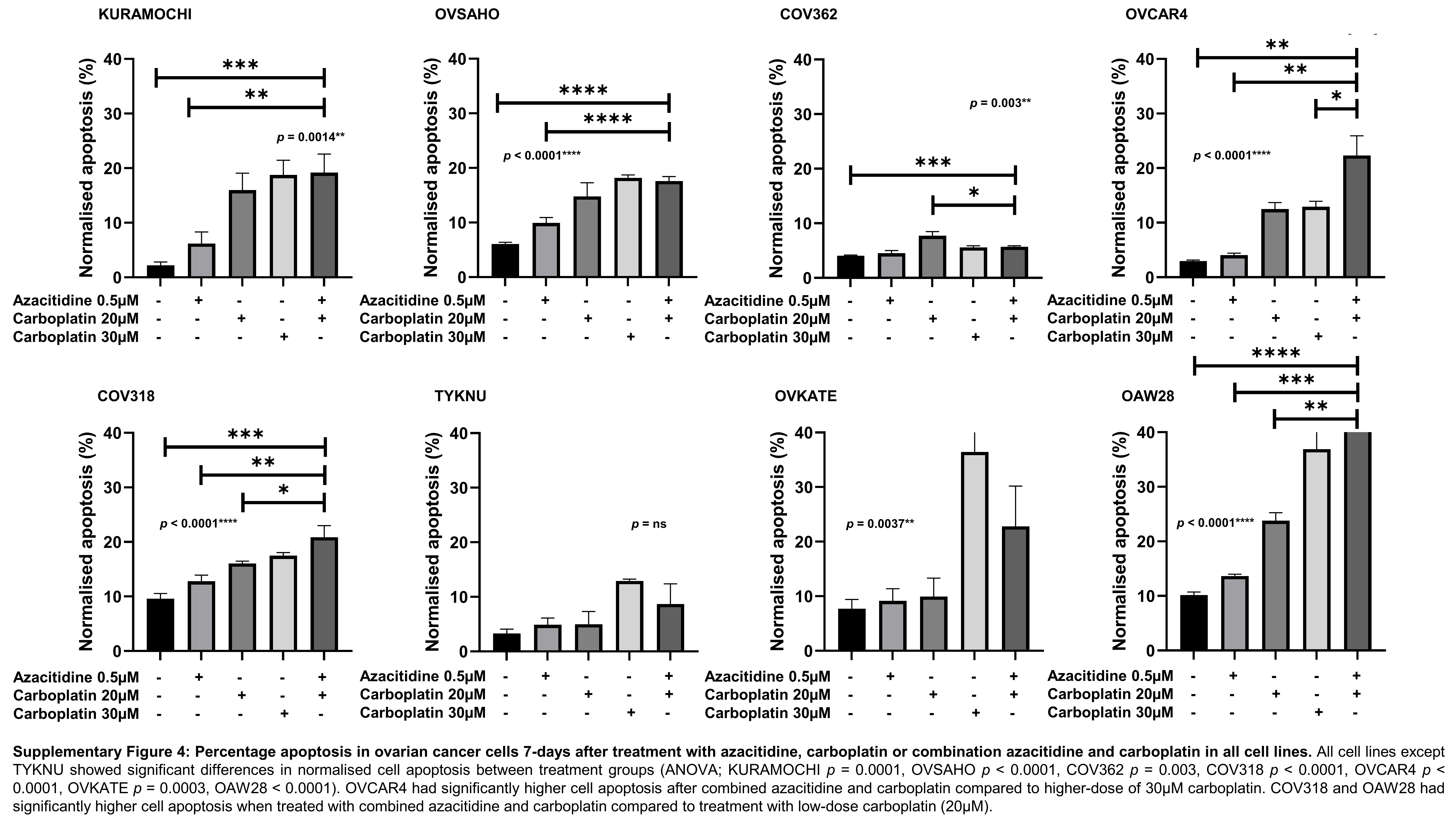

Supplement: Supplementary file 4 — Additional file 4. [file 12885_2022_9197_MOESM4_ESM.tif]

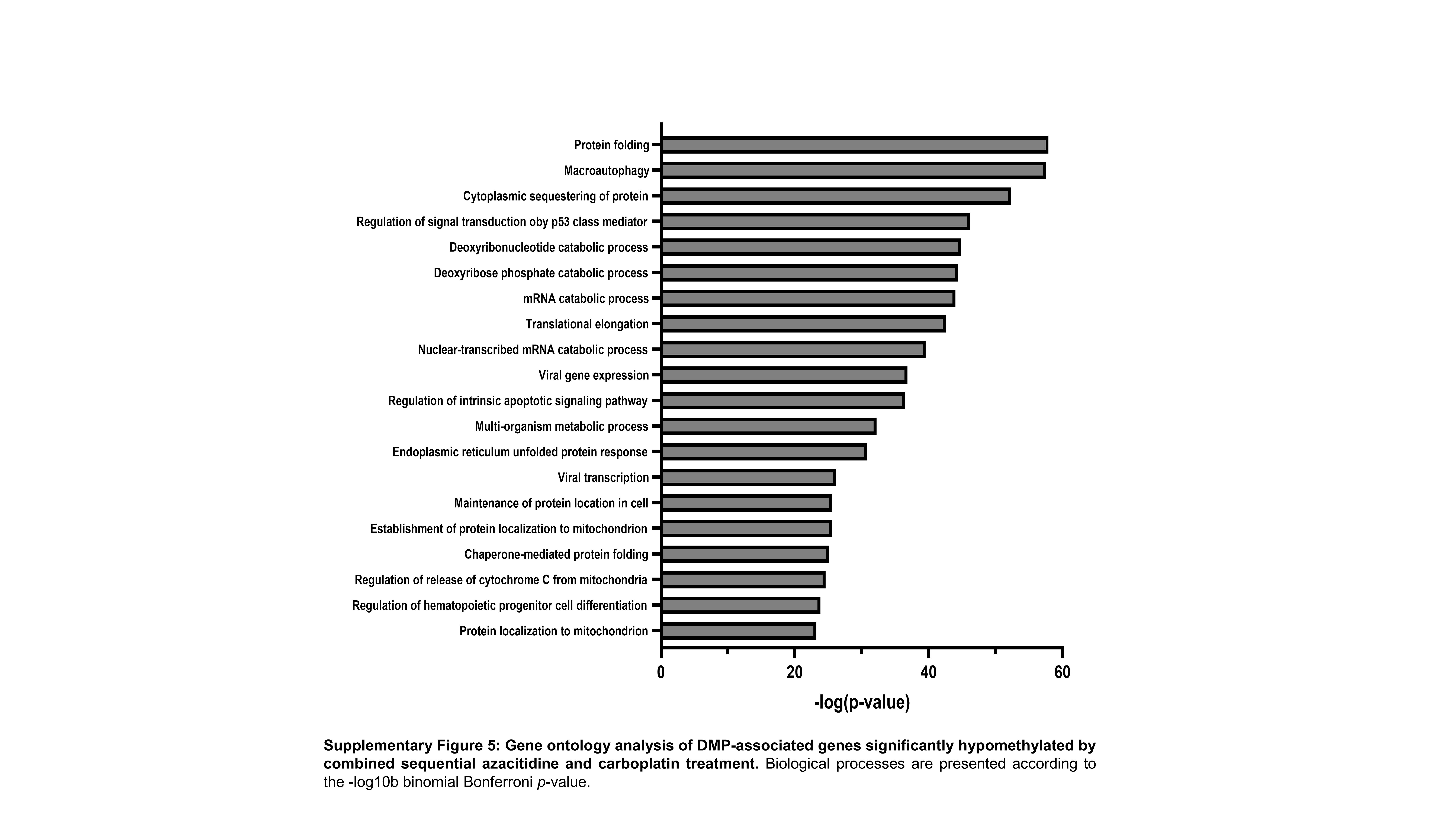

Supplement: Supplementary file 5 — Additional file 5. [file 12885_2022_9197_MOESM5_ESM.tif]

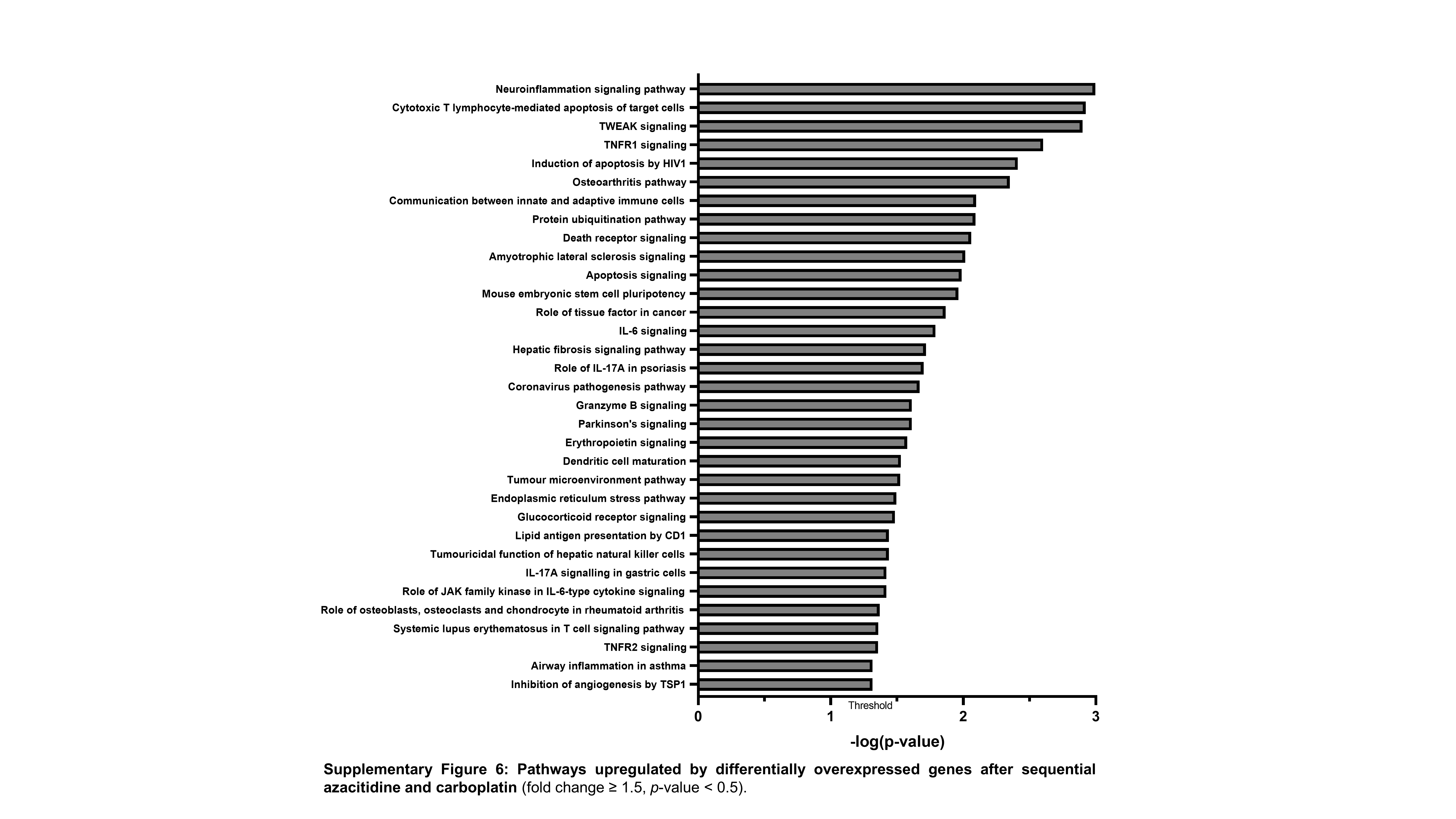

Supplement: Supplementary file 6 — Additional file 6. [file 12885_2022_9197_MOESM6_ESM.tif]
